# Surgery

**Published:** 1838-07

**Authors:** 


### SURGERY.

*A new Treatment in a Case of Anchylosis.* By J. R. Barton, M.D., Philadelphia.

[It is most gratifying to perceive, from the productions of the medical press of America, how completely the physicians and surgeons of that country are on a level with their brethren in Europe. In the boldness of their surgical operations they almost excel us, as the following most interesting case, as well as others which we have formerly cited, will evince.]

In the North American Medical and Surgical Journal for April, 1827, Dr. B. published an account of a new and successful operation at the hip, which had been undertaken for the twofold purpose of remedying a most serious deformity and lameness, and of *establishing an artificial joint,* as a substitute for the natural articulation, which had become obliterated by disease, terminating in true anchylosis.

In this case the neck of the femur was sawn through, and the distorted limb straightened. The wound of the soft parts was then healed, whilst the reunion of the divided bone was prevented by subjecting it, from time to time, to motion,—such as gentle rotation, flexion and extension, abduction and adduction. After continuing this treatment for a few weeks, the ends of the bone lost their disposition to unite, became obtunded and smooth, and were held attached to each other by provisional bands or ligaments, and in this manner forming an artificial joint, whose movements were regulated by all the principal muscles by which the original joint had been controlled.

The patient upon whom this operation was performed enjoyed the use of his artificial joint for six years; during which period he pursued a business (trunk-making) with great industry, earning for himself a comfortable subsistence, and a small annual surplus. Pecuniary losses, however, through the reverses of those in whose hands he had confided his means, sunk him into a state of despondency and desperation, followed by habits of intemperance. This, with all its train of evils, abuse of health, &c., was, no doubt, the cause of the change which afterwards took place in the artificial joint: it gradually became more and more rigid, and finally all motion ceased in the part.

The object of the treatment in the present case was different, but the means used were no less ingenious, and the results even more successful. The patient was a medical gentleman, Dr. Seaman Deas, now in active practice in Alabama. The following extracts give an abridged history of the case:

S. D., when a youth of about nine years of age, unluckily had his knee-joint involved in inflammation and suppuration so extensively as to occasion the destruction of the synovial membranes, the ligaments, cartilages, and, in short, every structure peculiarly appertaining to the joint. After a protracted suffering, he finally recovered with the loss of the joint; the tibia, femur, and patella having become united to each other in the form of a true anchylosis. The loss of the articulation of the knee, however, though a misfortune, did not constitute the *sadness* of his case: it was caused by the malposition of the limb; the leg having been flexed upon the thigh to a degree somewhat less than a right angle. Hence the only alternatives of which he could avail himself to aid in walking were, either to use crutches, or to employ a very high block-sole boot, and to lower his stature by flexing the sound limb, in order that both feet might reach the ground. The latter expedient he adopted. The long-continued pressure and weight of the body sustained by this defective limb, acting under such great mechanical disadvantages, had at length caused some projection of the instep, and other irregularities which it is unnecessary to particularize. This supposed irremediable condition of his limb, with all its ills, the young gentleman endured during the period of about sixteen years. In the meantime he graduated in medicine, and became a successful and highly respectable practitioner; but, as his professional labours increased, he found the condition of his limb to be an obstacle not only to his further success, but also a source of unceasing annoyance and vexation.

Dr. Deas having consulted Dr. Barton, and assented to the plan of cure proposed by him, the operation was performed on the 27th May, 1835, as follows:

Two incisions were made over the femur, just above the patella. The first commenced at a point opposite the upper and anterior margin of the external condyle of the femur, and, passing obliquely across the front of the thigh, terminated on the inner side. The second incision commenced also on the outer side, about two and a half inches above the first; and, passing likewise obliquely across the thigh, terminated with the other in an acute angle. By these incisions were divided the integuments, the tendon of the extensor muscles of the leg, at its insertion into the upper part of the patella, and some of the contiguous fibres of the rectus and crureus muscles themselves, a greater part of the vastus internus, and a portion of the vastus externus muscles. A flap, composed therefore of this structure, was elevated from the femur close to the condyles. The soft parts were next detached from the outer side of the bone, from the base of the flap toward the ham, by passing a knife over the circumference of it, so as to admit of the use of a saw. The flap then being turned aside, a triangular or wedge-like piece of the femur was easily removed by means of a small narrow-bladed saw, such as is used in the operation at the hip. This wedge of bone did not include the entire diameter of the femur at the point of section, so that a few lines of the posterior portion of the shaft of the bone remained yet undivided. By slightly inclining the leg backward, these yielded, and the solution was complete. This mode of effecting the lesion of the bone was designedly adopted, and constituted what I conceived to be a very important measure in the operation: important, because it rendered the popliteal artery free from the danger of being wounded by the action of the saw, and subsequently the interlocking of the fractured surfaces tended to retain the extremities of the divided bone in their positions until the harshness of their surfaces had been overcome, either by the absorption of their angles or by the deposition of new matter upon them; a change essential to the safety of the artery during the subsequent treatment of the case. Not a blood-vessel was opened which required either a ligature or compression. The operation, which lasted about five minutes, being thus ended, the reflected flap was restored to its place, the wound lightly dressed, and the patient was put to bed, lying on his back, with the limb supported upon a splint, *of an angle corresponding to that of the knee previous to the operation.* This position was maintained until it was believed that the asperities of the bone had become blunted, and were not likely by their pressure to cause ulceration of the artery beneath them. This first splint was then removed, and another, having the angle slightly obtuse, was substituted. In a few days a third splint, with the angle more obtuse than that of the second, supplied its place. Others, varying in degrees of angularity, in like manner came in their turn to support the limb, until it had attained a position almost straight: it was then unchangeably continued in that line until the contact surfaces of the bone had united, and securely fixed the limb in this the desired direction. . . .

At the end of about four months from the date of the operation, the patient stood erect, with both feet in their natural position, and the heels resting alike upon the floor, although a slight angle had been designedly left at the knee, in order that there might not be any necessity for throwing the limb out from the body in the act of walking, which is always the case when the knee is quite straight. After this period, the use of shoes of the ordinary shape was resumed, and the limb was daily exercised with increasing strength and usefulness.

[The sequel of the case is given in a letter from Dr. Deas himself, addressed to his accomplished surgeon, of which the following is the most important part:]

"I have the satisfaction and pleasure of saying to you now, that the operation you performed on my leg has been *completely* successful, and has more than realized my most sanguine anticipations. The small abscess which you dressed the day before we parted at Norfolk continued open, and threw out, from time to time, small pieces of bone until the August after, when the last piece was discharged; the orifice then closed, and I have suffered no material inconvenience from it since. From the January previous, however, I was going about and attending to my professional business; and early in the summer, when our sickly season commenced, I was on horseback daily, riding from thirty to fifty miles a day, without more than the ordinary fatigue or inconvenience. I am at present well, the wound sound, and I feel no other inconvenience in riding or walking than what arises from my knee-joint being stiff, which was the case before you performed the operation. I walk without a stick or other aid, with the sole of the foot to the ground, and, my friends tell me, with but a slight limp; and I have great pleasure in adding, that the leg and foot have increased considerably in size, so as now to be nearly equal to the other. When I think of what I was, and what I am; and that to your firmness, judgment, and skill, I am indebted for the happy change, I want words to express adequately all that I feel."

[We may almost envy the feelings of Dr. Barton on reading this letter. To save life, or, what is often more, to render a miserable life happy, by one bold and well-considered act; to have ourselves, and to permit to others no doubts as to the full amount of the benefit conferred; to be the object of such gratitude as only those can feel who have received so inestimable a blessing: these are the great and almost exclusive triumphs of SURGERY;—these are among the bright things in medical science which compensate for many of its difficulties, uncertainties, and cares; and ought to hallow its pursuit to the young as something conversant with man's diviner nature, and purify its practice from the low considerations which so pertinaciously cling to it as an art.]

*American Journal of Med. Sciences. Feb.* 1838.

---

*Treatment of Varicose Veins in the Pennsylvania Hospital.*

CASE I. By Dr. NORRIS. George K——, a German, aged 57 years, was admitted into the wards on the 19th of July, 1837, for varicose veins, from which he had suffered for several months. He had had a large ulcer caused by the veins, for which he had been treated in the city by bandages, &c. The ulcer was much reduced in size when he entered, and, after appropriate treatment, healed. On the 12th of August, Dr. Norris introduced two acupuncture needles,—one behind the vein, and the other through and through it in a line oblique to its axis, and surrounded both by a figure-of-eight ligatures. Little pain was caused by the operation. The limb was then elevated in a fracture-box; lead-water cloths applied; and the antiphlogistic treatment directed.

August 15th. The patient complains of no pain; little inflammation has occurred: ligature tightened, and treatment continued.—17th. Slight inflammation at the sutures: same treatment.—19th. The needles and ligatures were removed: some inflammation around the part, but none to any distance above or below.—24th. Inflammation increased; slight ulceration at the points where the needles entered: a poultice to the part, and antiphlogistic treatment continued.—Sept. 4th. The ulcers have healed; the vein perfectly obstructed: bandages and compress applied along the course of the vein.—7th. Allowed to walk about; has slight porriginous eruption: treated accordingly.—15th. Vein obliterated entirely; patient walks without feeling any inconvenience from it.—17th. Discharged, entirely well.

Within a few weeks the patient was seen, having no return of his complaint, and continuing constantly at work.

CASE II. By Dr. HARRIS. The subject of this case was 66 years of age, and had suffered from leg-ulcers for five years, which had been preceded, for two years, by a varicose condition of the veins.

Saturday, 3d February, the man was introduced into the amphitheatre, and stood upon the table. A fold of skin over the saphena vein, where it passes over the knee-joint, was held up by an assistant, and a bistoury passed through, dividing the skin and cellular substance down to the vein. A needle, armed with a ligature, was passed under the vein, which was drawn out, and a piece of it, half an inch in length, removed. A firm compress was placed above and below the orifice, the edges of the wound drawn together by adhesive plaster, and a bandage applied from the toes up to above the knee. The man was put to bed, and the limb placed in a long fracture-box. The bandage was not removed for a week. At the end of that time the wound was lightly touched with nitrate of silver; and, Wednesday, 21st, it had entirely healed. The veins are still visible through the skin, though considerably reduced in size. Bandage still applied.

*American Medical Examiner.* Nos. 5 and 9. 1838.

*A Statistical Account of Fractures, treated in the Pennsylvania Hospital, from its foundation in* 1751 *to* 1838. By J. M. Wallace, m.d., Resident Surgeon.

| I. Abstract of 197 Cases, treated from 1751 to 1800. Description of Fracture. | Number admitted. | Number cured. | No. of days required for cure. Max. | Min. | Average | Died from immediate effects of injury. | Died subsequently. | Terminated by Amputation. | Removed while under treatment. |
|---|---|---|---|---|---|---|---|---|---|
| Leg, Simple | 55 | 41 | 205 | 10 | 87 | 2 | 1 | 2 | 9 |
| Compound | 8 | 7 | 201 | 53 | 104 | 0 | 0 | 1 | 0 |
| Complicated | 9 | 9 | 591 | 77 | 213 | 0 | 0 | 0 | 0 |
| Thigh, Simple | 31 | 23 | 245 | 29 | 96 | 2 | 2 | 0 | 4 |
| Compound | 1 | 1 | 199 | 199 | 199 | 0 | 0 | 0 | 0 |
| Complicated | 10 | 10 | 314 | 42 | 118 | 0 | 0 | 0 | 0 |
| Arm, Simple | 34 | 24 | 162 | 21 | 69 | 3 | 0 | 0 | 7 |
| Complicated | 2 | 2 | 123 | 32 | 77 | 0 | 0 | 0 | 0 |
| Ribs | 20 | 13 | 195 | 11 | 50 | 2 | 0 | 0 | 5 |
| Skull | 9 | 4 | 205 | 35 | 137 | 3 | 1 | 0 | 1 |
| Clavicle | 8 | 7 | 42 | 16 | 29 | 0 | 0 | 0 | 1 |
| Jaw | 6 | 4 | 192 | 40 | 91 | 1 | 0 | 0 | 1 |
| Patella | 3 | 3 | 151 | 79 | 114 | 0 | 0 | 0 | 0 |
| Vertebræ | 1 | 1 | 507 | 507 | 507 | 0 | 0 | 0 | 0 |
| Pelvis | 1 | 0 | 0 | 0 | 0 | 0 | 0 | 0 | 1 |
| Sternum | 1 | 1 | 10 | 10 | 10 | 0 | 0 | 0 | 0 |
| Wrist | 1 | 1 | 255 | 255 | 255 | 0 | 0 | 0 | 0 |
| Fingers | 1 | 1 | 31 | 31 | 31 | 0 | 0 | 0 | 0 |
| Toes | 1 | 1 | 26 | 26 | 26 | 0 | 0 | 0 | 0 |
| Not designated | 4 | 1 | 72 | 72 | 72 | 2 | 0 | 0 | 1 |
| II. Do. 868 Cases, from 1800 to 1829. | | | | | | | | | |
| Leg, Simple | 249 | 216 | 217 | 10 | 70 | 9 | 12 | 0 | 12 |
| Compound | 10 | 3 | 220 | 83 | 136 | 6 | 1 | 0 | 0 |
| Complicated | 21 | 9 | 518 | 51 | 204 | 5 | 5 | 2 | 0 |
| Arm, Simple | 192 | 168 | 181 | 11 | 48 | 3 | 3 | 0 | 18 |
| Complicated | 8 | 5 | 66 | 25 | 48 | 3 | 0 | 0 | 0 |
| Thigh, Simple | 130 | 106 | 210 | 32 | 110 | 8 | 3 | 0 | 13 |
| Compound | 2 | 0 | 0 | 0 | 0 | 2 | 0 | 0 | 0 |
| Complicated | 18 | 8 | 418 | 77 | 158 | 7 | 2 | 0 | 1 |
| Clavicle, Simple | 61 | 58 | 84 | 9 | 36 | 0 | 0 | 0 | 3 |
| Complicated | 2 | 0 | 0 | 0 | 0 | 1 | 1 | 0 | 0 |
| Ribs, Simple | 45 | 38 | 70 | 6 | 34 | 2 | 0 | 0 | 5 |
| Complicated | 7 | 2 | 70 | 43 | 56 | 5 | 0 | 0 | 0 |
| Skull | 29 | 9 | 133 | 10 | 50 | 16 | 1 | 0 | 3 |
| Jaw | 22 | 15 | 136 | 11 | 34 | 1 | 1 | 0 | 5 |
| Patella | 12 | 12 | 144 | 33 | 73 | 0 | 0 | 0 | 0 |
| Vertebræ | 10 | 0 | 0 | 0 | 0 | 2 | 7 | 0 | 1 |
| Fingers, Simple | 10 | 6 | 63 | 20 | 43 | 1 | 0 | 0 | 3 |
| Compound | 1 | 1 | 74 | 74 | 74 | 0 | 0 | 0 | 0 |
| Elbow | 8 | 6 | 106 | 28 | 51 | 0 | 0 | 1 | 1 |
| Fibula | 6 | 5 | 175 | 51 | 99 | 0 | 1 | 0 | 0 |
| Nose | 4 | 2 | 22 | 9 | 15 | 0 | 0 | 0 | 2 |
| Shoulder | 4 | 3 | 58 | 26 | 39 | 0 | 0 | 0 | 1 |
| Scapula | 3 | 3 | 131 | 41 | 71 | 0 | 0 | 0 | 0 |
| Pelvis | 3 | 2 | 54 | 30 | 42 | 0 | 0 | 0 | 1 |
| Foot | 3 | 2 | 128 | 121 | 124 | 1 | 0 | 0 | 0 |
| Toes | 3 | 2 | 130 | 9 | 69 | 0 | 0 | 0 | 1 |
| Ankle | 3 | 2 | 66 | 42 | 54 | 1 | 0 | 0 | 0 |
| Hand | 2 | 2 | 48 | 23 | 35 | 0 | 0 | 0 | 0 |
| Not designated | 1 | 1 | 19 | 19 | 19 | 0 | 0 | 0 | 0 |

III. Abstract of 735 Cases, treated from 1829 to 1838.

| Description of Fracture. | Number admitted. | Number cured. | Number of days required for cure in adults. | | | Number of days required for cure in children under 18 years. | | | Died from the effects of injury. | Died subsequently | Ter. by amputation | Removed while under treatment. |
|---|---|---|---|---|---|---|---|---|---|---|---|---|
| | | | Max. | Min. | Aver. | Max. | Min. | Aver. | | | | |
| Leg, Simple | 172 | 150 | 516 | 14 | 74 | 88 | 44 | 64 | 11 | 5 | 1 | 5 |
| Compound | 37 | 21 | 374 | 54 | 141 | 103 | 79 | 91 | 8 | 5 | 2 | 1 |
| Complicated | 8 | 6 | 103 | 48 | 76 | 206 | 206 | 206 | 1 | 1 | 0 | 0 |
| Arm, Simple | 161 | 142 | 191 | 9 | 32 | 133 | 7 | 39 | 1 | 1 | 0 | 17 |
| Compound | 11 | 4 | 381 | 17 | 156 | 54 | 54 | 54 | 4 | 2 | 1 | 0 |
| Complicated | 10 | 6 | 81 | 42 | 54 | 0 | 0 | 0 | 2 | 0 | 1 | 1 |
| Clavicle, Simple | 75 | 63 | 68 | 8 | 31 | 39 | 15 | 23 | 0 | 0 | 0 | 12 |
| Complicated | 2 | 1 | 75 | 75 | 75 | 0 | 0 | 0 | 1 | 0 | 0 | 0 |
| Thigh, Simple | 73 | 60 | 254 | 40 | 92 | 137 | 14 | 66 | 7 | 0 | 0 | 6 |
| Compound | 10 | 1 | 0 | 0 | 0 | 45 | 45 | 45 | 4 | 3 | 2 | 0 |
| Complicated | 12 | 8 | 174 | 102 | 138 | 221 | 45 | 98 | 3 | 1 | 0 | 0 |
| Neck of Thigh | 4 | 0 | 0 | 0 | 0 | 0 | 0 | 0 | 0 | 0 | 0 | 4 |
| Skull, Simple | 31 | 13 | 156 | 14 | 57 | 115 | 18 | 66 | 15 | 1 | 0 | 2 |
| Compound | 3 | 1 | 0 | 0 | 0 | 69 | 69 | 69 | 2 | 0 | 0 | 0 |
| Complicated | 2 | 0 | 0 | 0 | 0 | 0 | 0 | 0 | 2 | 0 | 0 | 0 |
| Ribs, Simple | 35 | 29 | 116 | 4 | 29 | 0 | 0 | 0 | 3 | 1 | 0 | 2 |
| Complicated | 3 | 2 | 72 | 21 | 46 | 0 | 0 | 0 | 1 | 0 | 0 | 0 |
| Jaw, Simple | 17 | 11 | 58 | 17 | 38 | 126 | 33 | 79 | 4 | 1 | 0 | 1 |
| Compound | 2 | 2 | 0 | 0 | 0 | 126 | 25 | 77 | 0 | 0 | 0 | 0 |
| Patella, Simple | 12 | 11 | 151 | 45 | 70 | 0 | 0 | 0 | 0 | 0 | 0 | 1 |
| Compound | 1 | 1 | 140 | 140 | 140 | 0 | 0 | 0 | 0 | 0 | 0 | 0 |
| Fingers, Simple | 8 | 4 | 108 | 17 | 52 | 0 | 0 | 0 | 1 | 0 | 2 | 1 |
| Fibula, Simple | 6 | 5 | 47 | 25 | 33 | 0 | 0 | 0 | 0 | 0 | 0 | 1 |
| Complicated | 1 | 1 | 73 | 73 | 73 | 0 | 0 | 0 | 0 | 0 | 0 | 0 |
| Elbow, Simple | 8 | 8 | 74 | 19 | 51 | 44 | 37 | 40 | 0 | 0 | 0 | 0 |
| Compound | 3 | 3 | 336 | 55 | 197 | 0 | 0 | 0 | 0 | 0 | 0 | 0 |
| Scapula, Simple | 6 | 4 | 106 | 16 | 46 | 0 | 0 | 0 | 0 | 1 | 0 | 1 |
| Vertebræ | 4 | 0 | 0 | 0 | 0 | 0 | 0 | 0 | 1 | 3 | 0 | 0 |
| Pelvis, Compound | 1 | 0 | 0 | 0 | 0 | 0 | 0 | 0 | 1 | 0 | 0 | 0 |
| Ilium, Simple | 3 | 3 | 113 | 34 | 62 | 0 | 0 | 0 | 0 | 0 | 0 | 0 |
| Ankle, Simple | 2 | 1 | 39 | 39 | 39 | 0 | 0 | 0 | 0 | 0 | 0 | 1 |
| Compound | 2 | 1 | 463 | 463 | 463 | 0 | 0 | 0 | 1 | 0 | 0 | 0 |
| Sternum, Simple | 3 | 2 | 26 | 15 | 20 | 0 | 0 | 0 | 0 | 0 | 0 | 1 |
| Nose, Simple | 2 | 2 | 19 | 15 | 17 | 0 | 0 | 0 | 0 | 0 | 0 | 0 |
| Not designated | 5 | 4 | 225 | 50 | 107 | 0 | 0 | 0 | 1 | 0 | 0 | 0 |

*American Medical Examiner.* No. 2. 1838.

---

*Elm-bark Surgery.* By W. A. M'Dowall, m.d., of Fincastle, Virginia.

Under this title Dr. M'D. has given, in a recent journal, an account of the use of the bark of the slippery elm (*Ulmus fulva*) for bougies, tents, catheters, &c. It is not proposed that the new instruments should supersede the old, but to act as an adjuvant. Its advantages seem to be its ready fashioning by the surgeon to any shape or form, its pliability, smoothness, and great expansibility from imbibition of fluids. Dr. M'D. has employed it as a tent in abscesses and fistulæ, as a bougie in strictures, &c. He particularly recommends it in spasmodic stricture. As, probably, these instruments, or rather the materials for their fabrication, will reach this country, with other novelties, we must put on record the most important caution—and surely no less important drawback to its value—announced in the concluding sentence of this communication: "Some caution is necessary in using bougies or catheters of elm. Although this bark possesses a degree of tenacity surpassed by that of but few trees in the forest, yet, when seasoned and in a very dry state, it would be liable, in the hands of a careless or awkward operator, to break off in the urethra or bladder. To obviate this danger, it should be immersed in water, for a longer period when it is very dry, which will restore tenacity to its outer fibres." *The [Cincinnati] Western Journal of the Med. and Physical Sciences.* No. 43. *December,* 1837.

---

### ADDENDUM *to the "American and Colonial Journals."*

[Two or three of the journals from which the preceding extracts are made have not been previously noticed by us. Indeed, the great mass of materials of a scientific and practical character that press on us from all sides, has forced us to curtail our notices of a merely literary and critical character within narrower limits than we could wish. In addition to the *India Journal of Medical Science,* formerly noticed by us, (Vol. III. p. 192,) there has recently appeared, at Calcutta, a *Quarterly Medical Journal,* edited by Drs. Goodeve and O'Shaughnessy, professors in the Medical College of that place. This, like its predecessor, is an excellent work, and cannot fail to be useful to the profession in Europe as well as in India. Emanating, as it does, from the Calcutta Medical and Physical Society, and intended, we presume, to supersede the *Transactions* heretofore published by that body, it contains more elaborate and longer papers than the *Indian Journal,* and deals much less in the lighter literature which distinguishes our weekly journals. It is, in every respect, a very valuable publication, and such as might be expected from the great learning and high respectability of the editors. In both the Indian journals, the Selections from the British and foreign Journals are most copious, judiciously chosen, and well arranged. It is not a little complimentary to us that so very large a share of the extracts from the continental journals, in both publications, is taken from the "Selections" in this Review. We gladly accord to the editors the same privilege we claim for ourselves, in borrowing from our fellow-labourers; but we would suggest to them the same care which we invariably take of indicating the exact sources of our information. To a large proportion of the articles taken from us, the names of the *original* journals are alone subjoined, while that which was at the trouble and expense of selecting and translating them is omitted.

The *Medical Examiner* commenced at Philadelphia with the present year, and promises to be a valuable addition to the already large body of medical journals published in America. It is published *fortnightly,* and consists of one large royal 8vo. sheet, of small print and double columns, like our weekly journals. The Examiner resembles these also in the general character of its contents, and particularly in giving full reports of lectures; which, we believe, had not been previously done by any journal in the United States. It is edited by Drs. Biddle and Clymer; and costs three dollars per annum.

*The Boston Medical and Surgical Journal* has been established eight years, and has now reached its eighteenth volume. It is published weekly, and consists of one 8vo sheet of the common size. It is edited by Dr. J. V. C. Smith; and costs three dollars per annum. It is a highly respectable publication, and contains a good deal of useful matter; but the greater part of this is taken from foreign journals.]

